# Metabolic syndrome and its components among rheumatoid arthritis patients: A comprehensive updated systematic review and meta-analysis

**DOI:** 10.1371/journal.pone.0170361

**Published:** 2017-03-23

**Authors:** Jamal Hallajzadeh, Saeid Safiri, Mohammad Ali Mansournia, Maliheh Khoramdad, Neda Izadi, Amir Almasi-Hashiani, Reza Pakzad, Erfan Ayubi, Mark J. M. Sullman, Nahid Karamzad

**Affiliations:** 1 Department of Community Nutrition, Faculty of Nutrition Sciences and Food Technology, Shahid Beheshti University of Medical Sciences, Tehran, Iran; 2 Managerial Epidemiology Research Center, Department of Public Health, School of Nursing and Midwifery, Maragheh University of Medical Sciences, Maragheh, Iran; 3 Department of Epidemiology and Biostatistics, School of Public Health, Tehran University of Medical Sciences, Tehran, Iran; 4 Department of Epidemiology and Biostatistics, Faculty of Health, Kermanshah University of Medical Sciences, Kermanshah, Iran; 5 Department of Epidemiology, School of Public Health, Shahid Beheshti University of Medical Sciences, Tehran, Iran; 6 Department of Epidemiology and Reproductive Health, Reproductive Epidemiology Research Centre, Royan Institute for Reproductive Biomedicine, ACECR, Tehran, Iran; 7 Department of Epidemiology, Faculty of Health, Ilam University of Medical Sciences, Ilam, Iran; 8 Department of Public Health, School of Public Health, Zabol University of Medical Sciences, Zabol, Iran; 9 Driving Research Group, Cranfield University, Bedfordshire, United Kingdom; 10 Vice-Chancellery for Food and Drug, Maragheh University of Medical Sciences, Maragheh, Iran; Howard University, UNITED STATES

## Abstract

**Background:**

Estimating the current global prevalence of metabolic syndrome (MetS), and its components, among rheumatoid arthritis (RA) patients is necessary in order to formulate preventative strategies and to ensure there are adequate community resources available for these patients. Furthermore, the association between RA and MetS is controversial and has not previously been comprehensively assessed. Therefore, the present study aimed to: 1) determine the prevalence of MetS, and its components, among RA patients across the world 2) update the odds ratio of MetS in RA patients, compared to healthy controls, using a comprehensive systematic review and meta-analysis.

**Methods:**

International databases, including: the Web of Science, PubMed, Scopus, Embase, CINAHL and other relevant databases were searched to identify English language articles which reported the prevalence and risk of MetS in RA patients between January 2000 and August 2016. The meta-analysis only included studies which clearly described the time and location of the study, utilised adequate sampling strategies, and appropriate statistical analyses.

**Results:**

The meta-analyses of prevalence (70 studies [n = 12612]) and risk (43 studies [n = 35220]) of MetS in RA patients were undertaken separately. The overall pooled prevalence of MetS was 30.65% (95% CI: 27.87–33.43), but this varied from 14.32% (95% CI: 10.59–18.05) to 37.83% (95% CI: 31.05–44.61), based upon the diagnostic criteria used. The prevalence of MetS also varied slightly between males (31.94%, 95% CI: 24.37–39.51) and females (33.03%, 95% CI: 28.09–37.97), but this was not statistically significant. The overall pooled odds ratio (OR) of MetS in RA patients, compared to healthy controls, was 1.44 (95% CI: 1.20–1.74), but this ranged from 0.70 (95% CI: 0.27–1.76) to 4.09 (95% CI: 2.03–8.25), depending on the criteria used. The mean age and diagnostic criteria of MetS were identified as sources of heterogeneity in the estimated odds ratios between studies (P<0.05).

**Conclusions:**

According to the high prevalence of MetS in RA patients, and high risk of MetS, measuring metabolic syndrome in RA patients is strongly recommended. Furthermore, as high waist circumference (WC) is the most common metabolic syndrome component, more attention must be paid to nutrition and weight loss among those with RA.

## Introduction

Metabolic syndrome (MetS) is comprised of a group of risk factors for type 2 diabetes and cardiovascular diseases, including insulin resistance, abdominal obesity, dyslipidemia, blood pressure, and impaired fasting glucose[1]. The most common clinical manifestations of MetS include: abdominal obesity, hypertriglyceridaemia, reduced high-density lipoprotein cholesterol (HDL-C), hyperglycaemia, and high blood pressure (BP)[2]. MetS is responsible for a three-fold increase in the risk of atherosclerotic cardiovascular diseases (CVDs) and increased mortality from CVD, as well as all-causes, compared to the general population [3]. MetS is also associated with a fourfold increased relative risk of developing diabetes [4, 5]. There are eight commonly used definitions for MetS, but the National Cholesterol Education Programme-Adult Treatment Panel III (NCEP ATP III) and the International Diabetes Federation (IDF) definitions are the most commonly used [6]. These definitions have many similarities, but they differ on several components and on the cut-off points used (Table 1).

**Table 1:** Summary of the MetS definitions.

Definitions	WHO	NCEP-ATP III	IDF	EGIR	AACE	AHA/NHLBI	ATP III	JS 2009
**Number of Criteria**	Two or more of:	Three or more of:	Two or more of	Two or more of:	Obesity and two or more of:	Three or more of:	Three or more of:	Three or more of:
**Obesity**	BMI > 30 and/or WHR > 0.9 (men), WHR > 0.85 (women)	WC ≥ 102 cm (men), WC ≥ 88 cm (women	WC ≥ 94 cm men, WC ≥ 80 cm women	WC ≥ 94 cm (men, WC ≥80 cm (women)	WC ≥ 102 cm (men), WC ≥ 88 cm (women	BMI ≥ 30 kg/m2	WC ≥ 102 cm (men), WC ≥ 88 cm (women	Population- and country-specific definitions
**Blood pressure mmhg**	≥ 140/90	≥ 130/85 or treatment	≥130/≥85 or treatment	≥ 140/90	≥ 130/85 or treatment	≥130/85 mmHg or previous hypertension diagnosis	≥ 130/85 or treatment	≥ 130/85 or treatment
**Dyslipidmia:**								
**HDL-C**	≥ 35 mg/dL (0.9 mmol/L) in men or ≥ 39 mg/dL (≥ 1.0 mmol/L) in women	≥ 40 mg/dL (1.03 mol/L) in men, ≥ 50 mg/dL (1.29 mmol/L) in women, or treatment	≥ 40 mg/dL (1.03 mol/L) in men, ≥ 50 mg/dL (1.29 mmol/L) in women, or treatment	≥ 39 mg/dL (1.0 mmol/L) or treatment	≥ 40 mg/dL (1.03 mol/L) in men, ≥ 50 mg/dL (1.29 mmol/L) in women, or treatment	≥ 40 mg/dL (1.03 mol/L) in men, ≥ 50 mg/dL (1.29 mmol/L) in women	≥ 40 mg/dL (1.03 mol/L) in men, ≥ 50 mg/dL (1.29 mmol/L) in women	≥ 40 mg/dL (1.03 mol/L) in men, ≥ 50 mg/dL (1.29 mmol/L) in women, or treatment
**Triglycerides**	≥178 mg/dL(2.0 mmol/L) or treatment	≥150 mg/dL (1.7 mmol/L) or treatment	≥150 mg/dL (1.7 mmol/L) or treatment	≥150 mg/dL (1.7 mmol/L)	≥150 mg/dL (1.7 mmol/L) or treatment	≥150 mg/dL (1.7 mmol/L) or treatment	≥150 mg/dL (1.7 mmol/L)	≥150 mg/dL (1.7 mmol/L) or treatment
**Glucose Intolerance or Fasting Plasma Glucose**	≥110 mg/dL (6.1 mmol/l), DM, IGT, IR	≥100 mg/dL (5.6 mmol/L) or T2D	≥100 mg/dL (5.6 mmol/L) or T2D	≥110 mg/dL (6.1 mmol/L)	≥110 mg/dL (6.1 mmol/l), or treatment	≥100 mg/dL (5.6 mmol/L) or T2D	≥110 mg/dL (6.1 mmol/L)	≥100 mg/dL (5.6 mmol/L) or T2D

BMI = body mass index; JC = Joint Consensus; DM = diabetes mellitus; EGIR = European Group against Insulin Resistance; HDL-C = high-density lipoprotein cholesterol; IDF = International Diabetes Federation; IGT = impaired glucose tolerance; IR = insulin resistance; NCEP ATPIII = National Cholesterol Education Program Adult Treatment Panel; AACE = American Association of Clinical Endocrinologists; AHA/NHLBI = The American Heart Association / National Heart, Lung, and Blood Institute; JS = Joint Statement; T2 D, type II diabetes mellitus; WC = waist circumference; WHO = World Health Organization; WHR = waist hip ratio.

Therefore, although we could expect slight differences in prevalence rates, according to the criteria used in each study, genetic and geographical differences may also contribute to differences in the rates of MetS. For example, using the ATP III definition, Ford et al. reported the prevalence rate of metabolic syndrome in the USA to be 34.3% [3], while Tillin et al. reported the age-adjusted rates were 18.4% for men and 14.4% for women among Europeans, 28.8% for men and 31.8% for women in South Asians, and 15.5% for men and 23.4% for women in African-Caribbeans. Further, the prevalence rate was reported to be 15.7% in Taiwan, using the same criteria[7, 8].

Rheumatoid arthritis (RA) is a chronic inflammatory disorder of unknown etiology [9] that has a prevalence rate of approximately 0.5 to 1% [10]. Rheumatoid arthritis and metabolic syndrome are considered to be diseases with common traits that can increase the risk of cardiovascular disease[11], with previous research showing an association between the two[12]. Higher frequencies of insulin resistance and MetS have been reported in patients with RA [12, 13], with the frequency of MetS in RA patients ranging from 14 to 56% [14]. This variation can be explained by differences in the definition of MetS, along with differences in ethnicity, geographic area, study design, and study population. However, although many studies have reported a higher prevalence of MetS among RA patients, compared to the general population [15, 16], a number of studies have reported a higher prevalence of MetS in the healthy controls [2].

Research measuring the prevalence of MetS in RA patients has resulted in a wide range of estimates across the world. In addition, research measuring the prevalence of metabolic syndrome using a large sample size is rare. Furthermore, there have been very few meta-analyses on the prevalence of MetS in patients with rheumatoid arthritis [11]. Therefore, the present study aimed to: 1) determine the prevalence of MetS, and its components, in RA patients across the world 2) update the odds ratio of MetS in RA patients, compared to healthy controls, using a comprehensive systematic review and meta-analysis.

## Methods

### Search strategy and study selection

The current systematic review and meta-analysis was conducted according to PRISMA guidelines [17]. A systematic review was undertaken of English-language medical literature published between January 2000 and August 2016 to identify scientific papers reporting the prevalence and risk of metabolic syndrome and its components (i.e., waist circumference—WC, blood pressure—BP, high-density lipoprotein cholesterol -HDL-C, Triglycerides—TG, fasting blood sugar—FBS) among rheumatoid arthritis patients.

International databases, including: the Web of Science, Medline, Scopus, Embase, CABI, CINAHL, DOAJ, Index Medicus for Eastern Mediterranean Region-IMEMR and Google Scholar were searched using the following medical subject headings (MeSH): “Metabolic Syndrome”, “Dysmetabolic Syndrome”, “Cardiovascular Syndrome”, and “Insulin Resistance Syndrome”, combined with “Rheumatoid Arthritis”, “Prevalence”, “Odds Ratio”, “Comparative Cross-sectional Studies” and “case-control studies”. The search strategy for Medline was developed first and then adapted for the remaining databases. More detailed information regarding the search strategy is presented in Box 1. The grey literature were searched using Google Scholar, as recommended [18], using the abovementioned search strategy. An expert in this field was also consulted to identify additional papers.

**Box 1. Search strategy for MEDLINE (MeSH, Medical Subject Headings).**

1: Metabolic Syndrome [Text Word] OR Metabolic Syndrome [MeSH Terms]

2: Dysmetabolic Syndrome [Text Word] OR Dysmetabolic Syndrome [MeSH Terms]

3: Cardiovascular Syndrome [Text Word] OR Cardiovascular Syndrome [MeSH Terms]

4: Insulin Resistance Syndrome [Text Word] OR Insulin Resistance Syndrome [MeSH Terms]

5: 1 OR 2 OR 3 OR 4

6: Rheumatoid Arthritis [Text Word] OR Rheumatoid Arthritis [MeSH Terms]

7: 5 AND 6

8: Prevalence [Text Word] OR Prevalence [MeSH Terms]

9: Odds Ratio [Text Word] OR Odds Ratio [MeSH Terms]

10: Risk Ratio [Text Word] OR Risk Ratio [MeSH Terms]

11: Cross-Product Ratio [Text Word] OR Cross-Product Ratio [MeSH Terms]

12: 8 OR 9 OR 10 OR 11

13: Cross-sectional Studies [Text Word] OR Cross-sectional Studies [MeSH Terms]

14: Case-Control Studies [Text Word] OR Case-Control Studies [MeSH Terms]

15: Comparative cross-sectional Studies [Text Word] OR Comparative cross-sectional Studies [MeSH Terms]

16: 13 OR 14 OR 15

17: 7 AND 12 AND 16

All publications were categorized using Endnote X6. The title and abstract of identified publications were systematically screened and full texts were obtained for those which passed the initial screening. All full text publications were then independently evaluated by two reviewers (SS and JH) for inclusion in the review. Disagreements between the reviewers were resolved by consensus using a third expert (MN). In this study, blinding and task separation were also applied to study selection.

All English language observational (cross-sectional and comparative cross-sectional) studies on the prevalence of metabolic syndrome were included in the current study if they clearly described the date of data collection and study location, used appropriate sampling strategies, and conducted appropriate statistical analyses. Case studies and letters to the editor were excluded, along with systematic reviews or meta-analyses. Lastly, studies undertaken on patients with other disorders were also excluded.

### Data extraction and quality assessment

Study characteristics (first author’s name, date of publication, and country of origin), participant characteristics (gender, age, and sample size), and MetS prevalence (based on the different criteria) were extracted using the full text reviews. The quality of each included study was also assessed using the STROBE checklist [19].

### Statistical analysis

All statistical analyses were undertaken using Review Manager (RevMan) Version 5.3. (Copenhagen: The Nordic Cochrane Centre, The Cochrane Collaboration, 2014). The prevalence of metabolic syndrome, and its five components, among rheumatoid arthritis patients were pooled using a random-effects model and presented in a forest plot. The odds ratios for metabolic syndrome in rheumatoid arthritis patients, based upon the different diagnostic criteria, in comparative cross-sectional studies were also pooled using a random-effects model and presented in a forest plot. Statistical heterogeneity was assessed using the I^2^ index and a random-effects model was used when the I^2^ index was > 0.6. Stata software version 13 (Stata Corp, College Station, TX, USA) was used to determine which factors were responsible for any observed heterogeneity using meta-regression. Publication bias, with regards to the ORs between MetS and RA was assessed using a Funnel plot and Begg's correlation test [20].

## Results

**Fig 1:**
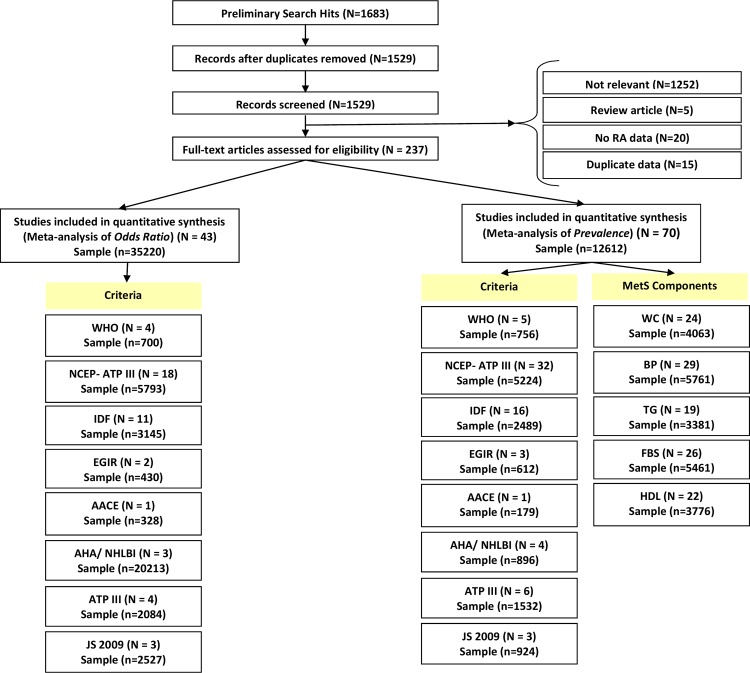
Flow diagram of the study selection process.

After removing duplicates, our primary search found 237 relevant articles. Following the exclusion of all non-eligible studies a total of 70 cross-sectional studies and 43 comparative cross-sectional studies, from 25 countries, were retained to estimate the prevalence and risk of metabolic syndrome among RA patients. The details of our study selection method are shown in Fig 1. The majority of the studies reporting MetS prevalence (55 studies) included both male and female patients who were aged >18 years. The lowest and highest prevalence of MetS in rheumatoid arthritis patients reported were 10.6% and 55.5%, respectively. More detailed information about each included studies can be found in Table 2.

**Table 2:** Worldwide prevalence (95% CI) of metabolic syndrome in rheumatoid arthritis patients.

First Author	Country	Criteria	DOP	Age Range	Mean Age	Gender	N. of RA Patients			Prevalence of MetS in RA Patients (%)			Reference
							Total	Male	Female	Total	Male	Female	
Lee SH	Korea	AHA/NHLBI	2016	≥12	63.6	Both	598	110	488	36.4	34.5	36.9	[37]
Hugo M	France	IDF	2016	18–75	57.6	Both	57	15	42	24.0	25.0	24.0	[38]
Zafar ZA	Pakistan	NCEP-ATP III	2016	20–60	43.8	Both	384	97	277	31.3	18.5	35.5	[35]
Oliveira BMGB	Brazil	NCEP-ATP III	2016	-	55.5	Female	107	-	107	51.4	-	51.4	[24]
Oliveira BMGB	Brazil	IDF	2016	-	55.5	Female	107	-	107	53.4	-	53.4	[24]
Muller R	Estonia	NCEP-ATP III	2016	-	51.6	Both	91	66	25	35			[33]
Dihingia P	India	NCEP-ATP III	2016	>12	41.5	Both	72	6	66	16.7			[39]
Ghazaly AHAH	Egypt	ATP III	2015	≥18	40.7	Both	80	13	67	50.0	53.8	49.2	[40]
Salamon L	Croatia	ATP III	2015	52–68	59	Both	583	100	483	43.1	40.0	43.7	[41]
Tanayakom P	Thailand	NCEP-ATP III	2015	-	59	Both	267	31	236	16.1	12.9	16.5	[42]
Parra-Salcedo F	Mexico	AHA/NHLBI	2015	-	38.1	Both	160	18	142	28.0			[43]
Parra-Salcedo F	Mexico	IDF	2015	-	38.1	Both	160	18	142	18.0			[43]
Parra-Salcedo F	Mexico	NCEP-ATP III	2015	-	38.1	Both	160	18	142	24.0			[43]
Craciun L	Romania	IDF-AHA	2014	32–79	55.2	Both	51	7	77	19.0	10.52	82.47	[23]
Craciun L	Romania	NCEP-ATP III	2014	32–79	55.2	Both	51	7	77	23.0			[23]
Craciun L	Romania	IDF	2014	32–79	55.2	Both	51	7	77	18.0			[23]
Craciun L	Romania	AHA	2014	32–79	55.2	Both	51	7	77	14.0			[23]
Bilecik NA	Turkey	IDF	2014	24–65	52.0	Female	100	-	100	33.0	-	33.0	[44]
Bilecik NA	Turkey	NCEP-ATP III	2014	24–65	52.0	Female	100	-	100	27.0	-	27.0	[44]
Özmen M	Turkey	NCEP-ATP III	2014	-	51.0	Both	52	15	37	17.30			[45]
Özmen M	Turkey	WHO	2014	-	51.0	Both	52	15	37	28.80			[45]
Kumar BS	India	IDF	2014	≥18	46.0	Both	54	6	48	29.0			[46]
Kumar BS	India	NCEP-ATP III	2014	≥18	46.0	Both	54	6	48	31.0			[46]
Abourazzak FE	Morocco	IDF	2014	>16	49.0	Both	179	22	157	30.7			[26]
Abourazzak FE	Morocco	NCEP-ATP III	2014	>16	49.0	Both	179	22	157	29.0			[26]
Abourazzak FE	Morocco	AACE 2003	2014	>16	49.0	Both	179	22	157	24.6			[26]
Salinas MJH	Argentina	ATP III	2013	-	55.5	Both	409	69	340	30.0	62.0	23.8	[47]
Salinas MJH	Argentina	IDF	2013	-	55.5	Both	409	69	340	35.0			[47]
Abdul-Qahar	Iraq	NCEP-ATP III	2013	-	46.9	Both	203	41	162	51.2	12.0	92.0	[48]
Rostam S	Morocco	NCEP-ATP III-2004	2013	-	49.0	Both	120	10	110	30.8	10.0	32.7	[49]
Rostam S	Morocco	NCEP-ATP III-2001	2013	-	49.0	Both	120	10	110	24.6			[49]
Rostam S	Morocco	WHO	2013	-	49.0	Both	120	10	110	20.0			[49]
Rostam S	Morocco	IDF	2013	-	49.0	Both	120	10	110	48.6			[49]
Rostam S	Morocco	EGIR	2013	-	49.0	Both	120	10	110	18.0			[49]
Rostam S	Morocco	JC 2009	2013	-	49.0	Both	120	10	110	32.3			[49]
Lee SG	Korea	NCEP-ATP III	2013	22–76	50.6	Female	84	-	84	19.0	-	19.0	[34]
Ormseth MJ	USA	ATP III	2013	≥18	54.0	Both	162	18	144	36.0			[50]
Karakoc	Turkey	IDF	2012	-	49.8	Both	54	7	47	42.6			[51]
Manka V	Slovakia	IDF	2012	≥18	58.8	Both	87	4	83	48.3			[52]
Manka V	Slovakia	NCEP-ATP III	2012	≥18	58.8	Both	87	4	83	44.8			[52]
Manka V	Slovakia	AHA/NHLBI	2012	≥18	58.8	Both	87	4	83	47.1			[52]
Cunha VR Da	Brazil	NCEP-ATP III	2012	≥18	56.8	Both	283	50	233	39.2			[53]
Goshayeshi L	Iran	NCEP-ATP III	2012	-	45.5	Both	120	14	106	45.2			[21]
Bkaer JF	USA	IDF	2012	18–85	49.5	Both	499	83	416	10.6			[54]
Crowson CS	USA	NCEP-ATP III	2011	≥18	58.8	Both	232	58	174	33.0	36.0	32.0	[31]
Sahaberi M	Iran	IDF	2011	-	45.5	Both	120	14	106	30.8	28.6	41.5	[55]
Sahaberi M	Iran	NCEP-ATP III	2011	-	45.5	Both	120	14	106	45.2	28.6	37.7	[55]
Karimi M	Iran	NCEP	2011	≥18	48.3	Female	92	-	92	27.2	-	27.2	[22]
Karimi M	Iran	WHO	2011	≥18	48.3	Female	92	-	92	19.6	-	19.6	[22]
Mok CC	Hong Kong	JS 2009	2011	≥18	53.3	Both	699	133	566	20.0			[56]
Dao HH	Vietnam	IDF	2010	26–73	56.3	Female	105	-	105	40.9	-	40.9	[57]
Dao HH	Vietnam	NCEP-ATP III 2004	2010	26–73	56.3	Female	105	-	105	32.4	-	32.4	[57]
Dao HH	Vietnam	NCEP-ATP III 2001	2010	26–73	56.3	Female	105	-	105	24.7	-	24.7	[57]
Dao HH	Vietnam	JS 2009	2010	26–73	56.3	Female	105	-	105	32.4	-	32.4	[57]
Dao HH	Vietnam	WHO	2010	26–73	56.3	Female	105	-	105	19.0	-	19.0	[57]
Dao HH	Vietnam	EGIR	2010	26–73	56.3	Female	105	-	105	16.2	-	16.2	[57]
Raterman H G	Netherlands	NCEP	2010	50–75	62.1	Both	236	79	157	19.9			[58]
Solomon A	South Africa	NCEP-ATP III	2010	-	27.2	Both	291	32	259	31.3			[59]
Solomon B	South Africa	NCEP-ATP III	2010	-	27.2	Both	335	65	270	20.3			[59]
Giles J	USA	NCEP-ATP III	2010	45–84	61	Both	131	51	80	36.0			[60]
Santos MJ	Portugal	ATP III	2010	≥18	49.2	Female	98		98	25.5			[61]
Toms TE	UK	IDF	2009	55.5–69.6	63.1	Both	387	105	282	45.3	52.7	42.6	[25]
Toms TE	UK	NCEP-ATP III 2004	2009	55.5–69.6	63.1	Both	387	105	282	40.1	42.5	39.2	[25]
Toms TE	UK	NCEP-ATP III 2001	2009	55.5–69.6	63.1	Both	387	105	282	38.3	40.0	37.7	[25]
Toms TE	UK	WHO	2009	55.5–69.6	63.1	Both	387	105	282	19.4	25.5	17.2	[25]
Toms TE	UK	EGIR	2009	55.5–69.6	63.1	Both	387	105	282	12.1	22.6	8.2	[25]
Chung CP	USA	WHO	2008	≥18	59	Both	66	18	48	42.0			[29]
Zonana-Nacach A	Mexico	NCEP-ATP III	2008	-	42.9	Both	107			18.7			[30]
Karvounaris SA	Greece	ATP III	2007	≥18	63.0	Both	200	53	147	44.0	39.6	45.6	[32]
Montagna G La	Italy	NCEP-ATP III	2007	-	53.8	Both	45	3	42	55.5			[62]

The estimated pooled prevalence, with 95% confidence interval (the diamond below the graph shows the pooled prevalence and the horizontal lines define the reported 95% confidence interval in each study) are presented in graphs by gender and by MetS definition.

### Total MetS prevalence in RA patients by gender

**Fig 2:**
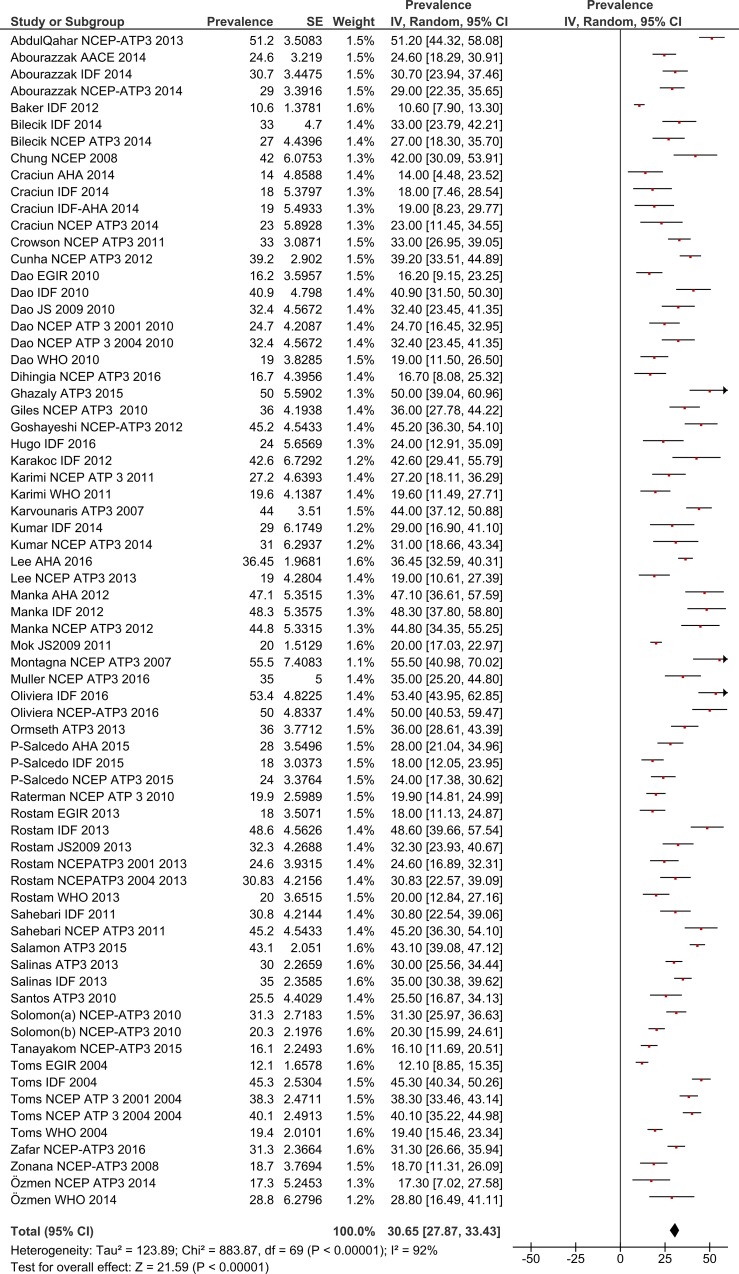
Forest plot of MetS prevalence in RA Patients.

**Fig 3:**
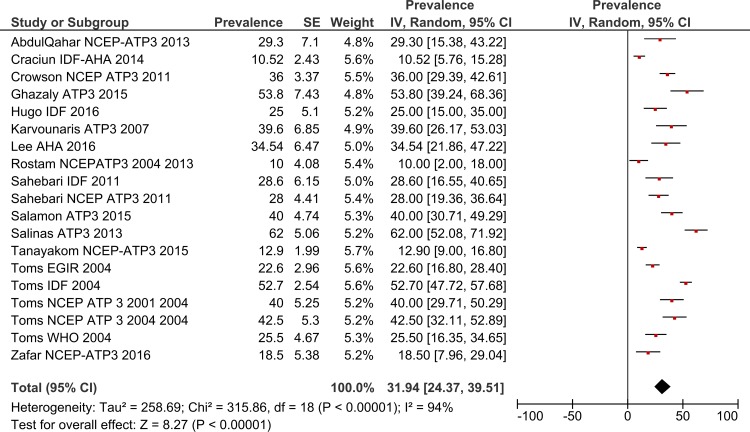
Forest plot of MetS prevalence among male RA Patients.

**Fig 4:**
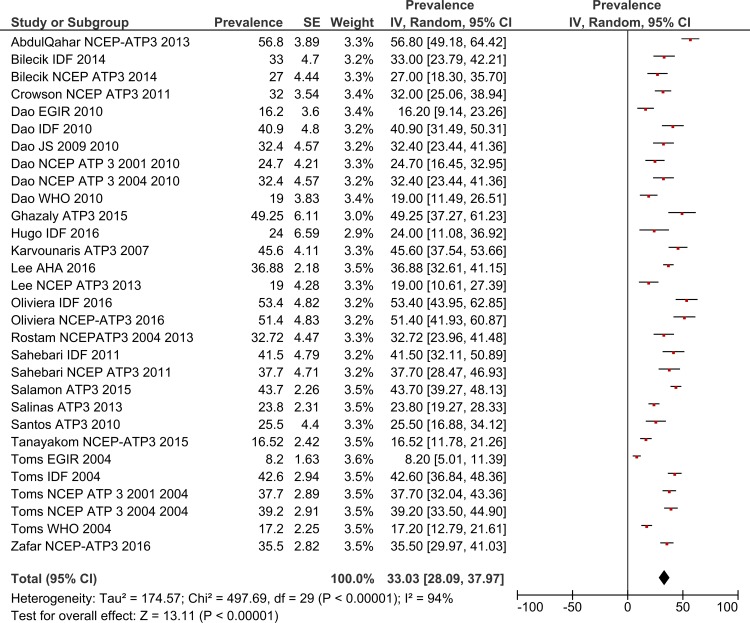
Forest plot of MetS prevalence among female RA Patients.

Using a random effects model, the estimated worldwide prevalence rate of MetS among RA patients was 30.65% (95% CI: 27.87–33.43) (Fig 2). In addition, information on the prevalence of MetS by gender was available from 19 studies for males and 30 for females. The prevalence rates among males was 31.94% (95% CI: 24.37–39.51) and for females this was 33.03% (95% CI: 28.09–37.97) (Figs 3 and 4).

### MetS prevalence in RA patients by criteria/definition

**Fig 5:**
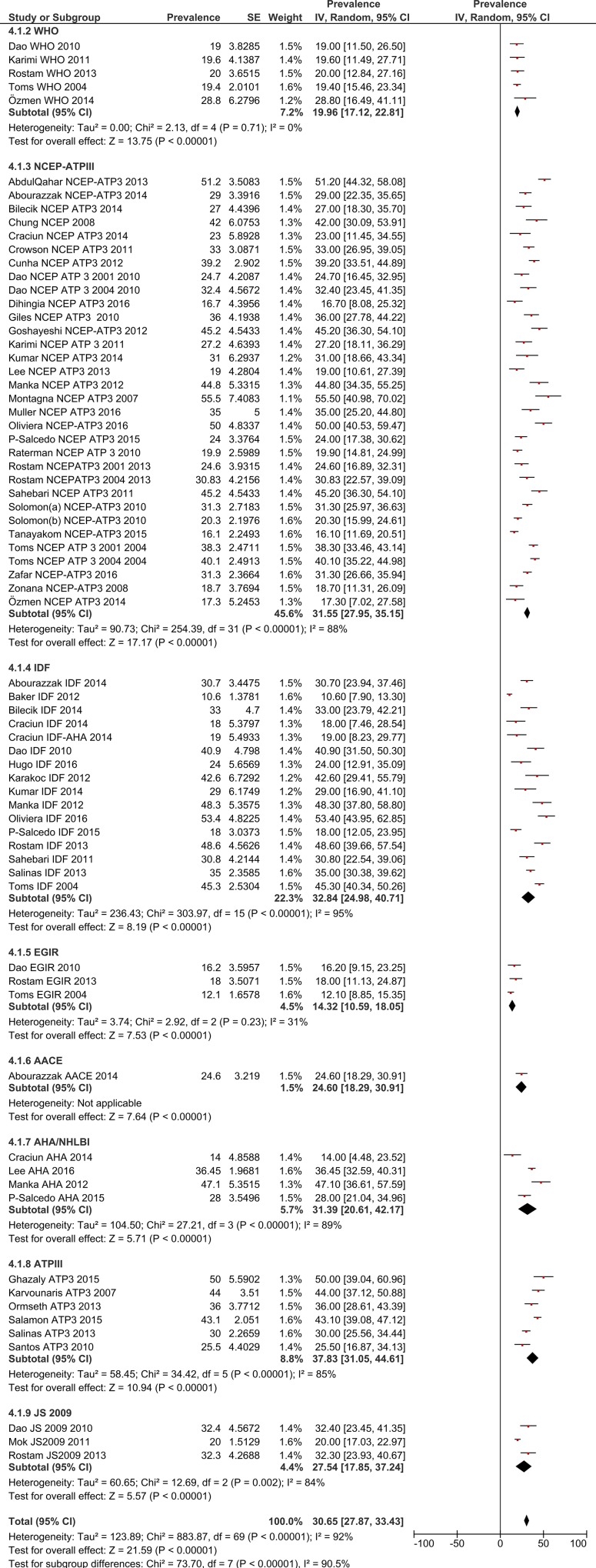
Forest plot of MetS prevalence among RA Patients by definition/criteria.

The pooled MetS prevalence rates for the eight definitions are: **WHO**—19.96% (95% CI: 17.12–22.81), **NCEP/ATP III**—31.55% (95% CI: 27.95–35.15), **IDF**—32.84% (95% CI: 24.98–40.71), **EGIR**—14.32% (95% CI: 10.59–18.05), **ACCE**—24.6% (95% CI: 19.29–30.91), **AHA/NHBI—**31.39% (95% CI: 20.61–42.17), **ATP III**—37.83% (95% CI: 31.05–44.61) and **JS 2009**–27.54 (95% CI: 17.85–37.24) (Fig 5).

### MetS prevalence in rheumatoid arthritis patients by MetS component

The MetS components of FBS, HDL-C, BP, Triglyceride and Waist Circumstance (WC) were reported by 26, 22, 29, 19 and 24 studies, respectively. The pooled MetS prevalence rates, by component, were: **FBS**—19.47% (95% CI: 15.69–23.25), **HDL**—41.78% (95% CI: 28.73–54.84), **BP**—48.65% (95% CI: 41.03–56.26), **Triglyceride**—28.43% (95% CI: 22.3–34.57) and **WC**—52.63 (95% CI: 43.76–61.5) (S 1–5 Appendix).

### Risk of MetS in rheumatoid arthritis patients by criteria/definition

**Fig 6:**
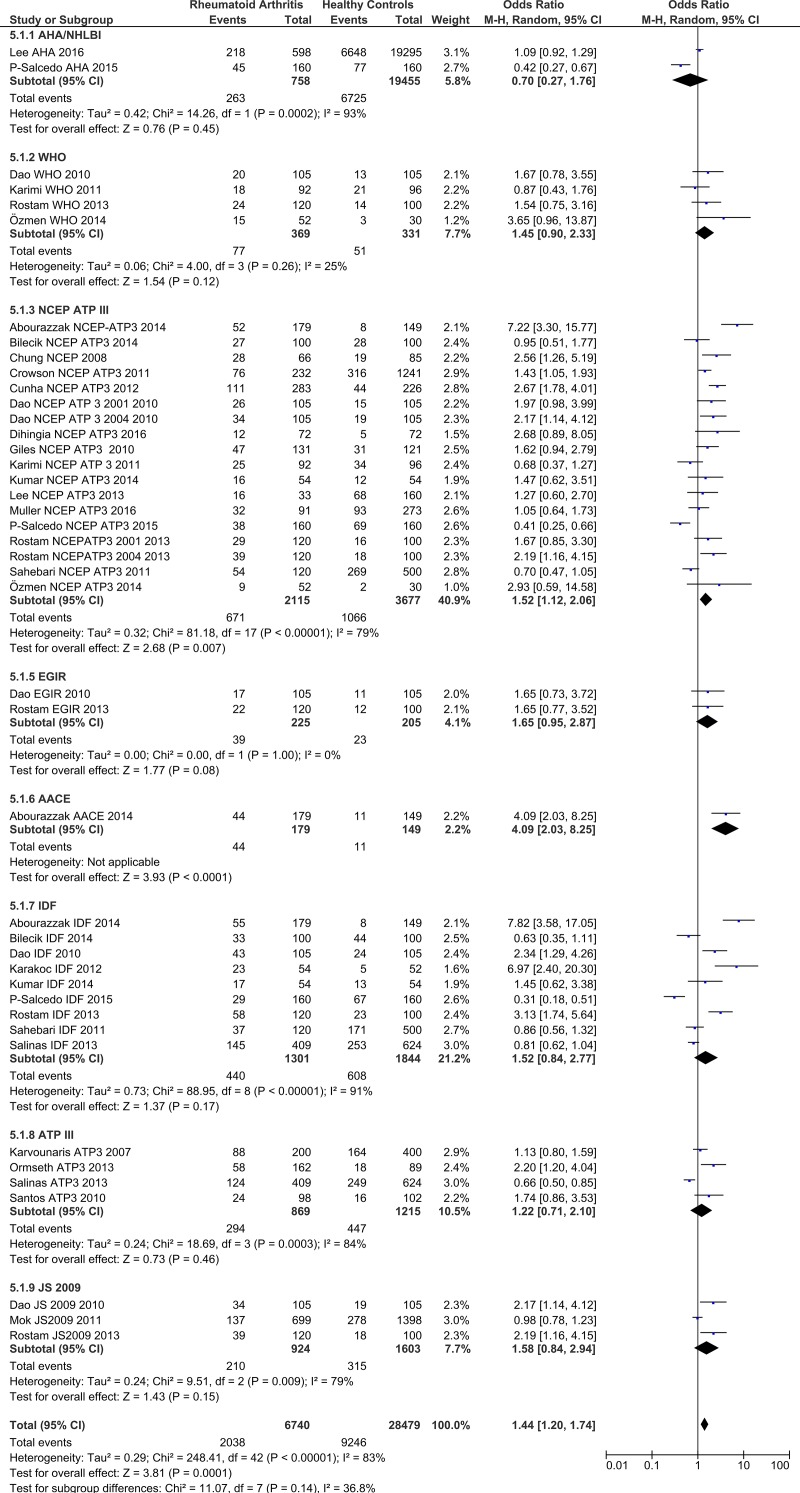
Forest plot of MetS risk among RA patients by definition/criteria.

In this section the prevalence of MetS in RA patients and among healthy controls were compared (Table 3). The pooled estimates identified a significant positive association between rheumatoid arthritis and the risk of MetS (OR = 1.44; 95% CI: 1.20–1.74). The odds ratios for MetS in rheumatoid arthritis patients, according to the definition used, were: **WHO**—OR = 1.45 (95% CI: 0.9–2.33), **NCEP/ATP III**—OR = 1.52 (95% CI: 1.12–2.06), **IDF**—OR = 1.52 (95% CI: 0.84–2.77), **EGIR**—OR = 1.65 (95% CI: 0.95–2.87), **ACCE**—OR = 4.09 (95% CI: 2.03–8.25), **AHA/NHBI**—OR = 0.7 (95% CI: 0.27–1.76), **ATP III**—OR = 1.22 (95% CI: 0.71–2.1), and **JS 2009**—OR = 1.58 (95% CI: 0.84–2.94) (Fig 6).

**Table 3:** Worldwide prevalence (95% CI) of metabolic syndrome in rheumatoid arthritis patients compared to healthy controls.

First Author	Country	Criteria	DOP	Gender	N. RA Patients						N. Healthy Controls						Reference
					Mean Age	Age Range	Male	Female	Total		Mean Age	Age Range	Male	Female	Total		
									N.	MetS Prev. (%)					N.	MetS Prev. (%)	
Lee SH	Korea	AHA/NHLBI	2016	Both	63.6	-	110	488	598	36.45	58.4	-	8114	11181	19295	34.45	[37]
Muller R	Estonia	NCEP-ATP III	2016	Both	51.6	-	66	25	91	35.16	51.5	-	75	198	273	34.06	[33]
Dihingia P	India	NCEP-ATP III	2016	Both	41.5	-	6	66	72	16.66	-	-	-	-	72	6.94	[39]
Parra-Salcedo F	Mexico	AHA/NHLBI	2015	Both	38.1	-	18	142	160	28.12	38.0	-	18	142	160	4.81	[43]
Parra-Salcedo F	Mexico	IDF	2015	Both	38.1	-	18	142	160	18.12	38.0	-	18	142	160	4.18	[43]
Parra-Salcedo F	Mexico	NCEP-ATP III	2015	Both	38.1	-	18	142	160	23.75	38.0	-	18	142	160	4.31	[43]
Bilecik NA	Turkey	IDF	2014	Female	52.0	24–65	0	100	100	33.0	51.0	27–65	0	100	100	44.0	[44]
Bilecik NA	Turkey	NCEP-ATP III	2014	Female	52.0	24–65	0	100	100	27.0	51.0	27–65	0	100	100	28.0	[44]
Özmen M	Turkey	NCEP-ATP III	2014	Both	51.0	-	15	37	52	17.30	48.0	-	9	21	30	6.60	[45]
Özmen M	Turkey	WHO	2014	Both	51.0	-	15	37	52	28.84	48.0	-	9	21	30	10.0	[45]
Kumar BS	India	IDF	2014	Both	46.0	-	6	48	54	31.48	45.4	-	6	48	54	24.07	[46]
Kumar BS	India	NCEP-ATP III	2014	Both	46.0	-	6	48	54	29.62	45.4	-	6	48	54	22.22	[46]
Abourazzak FE	Morocco	IDF	2014	Both	49.0	-	22	157	179	30.72	51.0	-	23	126	149	5.36	[26]
Abourazzak FE	Morocco	NCEP-ATP III	2014	Both	49.0	-	22	157	179	29.05	51.0	-	23	126	149	5.36	[26]
Abourazzak FE	Morocco	AACE 2003	2014	Both	49.0	-	22	157	179	24.58	51.0	-	23	126	149	7.38	[26]
Salinas MJH	Argentina	ATP III	2013	Both	55.5	-	69	340	409	30.31	57.3	-	103	521	624	39.90	[47]
Salinas MJH	Argentina	IDF	2013	Both	55.5	-	69	340	409	35.45	57.3	-	103	521	624	40.54	[47]
Chung CP	Usa	NCEP-ATP III	2008	Both	59.0	43–59	18	48	66	42.42	52.0	44–58	30	55	85	22.35	[29]
Dao HH	Vietnam	WHO	2010	Female	56.3	26–73	0	105	105	19.04	55.7	25–72	56	49	105	12.35	[57]
Dao HH	Vietnam	IDF	2010	Female	56.3	26–73	0	105	105	40.95	55.7	25–72	56	49	105	22.85	[57]
Dao HH	Vietnam	NCEP-ATP III	2010	Female	56.3	26–73	0	105	105	24.76	55.7	25–72	56	49	105	14.28	[57]
Dao HH	Vietnam	NCEP-ATP III	2010	Female	56.3	26–73	0	105	105	32.38	55.7	25–72	56	49	105	18.09	[57]
Dao HH	Vietnam	EGIR	2010	Female	56.3	26–73	0	105	105	16.19	55.7	25–72	56	49	105	10.47	[57]
Dao HH	Vietnam	JS2009	2010	Female	56.3	26–73	0	105	105	32.38	55.7	25–72	56	49	105	18.09	[57]
Karimi M	Iran	NCEP-ATP III	2011	Both	48.3	-	-	-	92	27.17	42.2	-	-	-	96	35.41	[22]
Rostam S	Morocco	WHO	2013	Both	49.0	-	10	110	120	20.00	48.5	-	10	90	100	14.00	[49]
Rostam S	Morocco	IDF	2013	Both	49.0	-	10	110	120	48.60	48.5	-	10	90	100	23.00	[49]
Rostam S	Morocco	NCEP-ATP III	2013	Both	49.0	-	10	110	120	24.16	48.5	-	10	90	100	16.00	[49]
Rostam S	Morocco	NCEP-ATP III	2013	Both	49.0	-	10	110	120	32.50	48.5	-	10	90	100	18.0	[49]
Rostam S	Morocco	EGIR	2013	Both	49.0	-	10	110	120	18.33	48.5	-	10	90	100	12.00	[49]
Rostam S	Morocco	JS2009	2013	Both	49.0	-	10	110	120	32.50	48.5	-	10	90	100	18.0	[49]
Crowson CS	Usa	NCEP-ATP III	2011	Both	58.8	-	58	174	232	32.75	63.9	-	560	681	1241	25.46	[31]
Cunha VR da	Brazil	NCEP-ATP III	2012	Both	56.8	-	50	233	283	39.22	44.5	-	34	192	226	19.46	[53]
Giles JT	Usa	NCEP-ATP III	2010	Both	61.0	-	51	80	131	35.87	63.0	-	70	51	121	25.61	[60]
Sahebari M	Iran	NCEP-ATP III	2011	Both	45.5	-	14	106	120	45.0	45.6	-	69	431	500	53.8	[55]
Sahebari M	Iran	IDF	2011	Both	45.5	-	14	106	120	30.83	45.6	-	69	431	500	34.2	[55]
Karakoc M	Turkey	IDF	2012	Both	49.7	-	7	47	54	42.59	47.0	-	43	9	52	9.61	[51]
Santos MJ	Portugal	ATP III	2010	Female	49.2	-	0	98	98	24.48	47.7	-	0	102	102	15.68	[61]
Mok CC	Hong Kong	JS2009	2011	Both	53.3	-	133	566	699	19.59	52.9	-	266	1132	1398	19.88	[56]

### Publication bias

**Fig 7:**
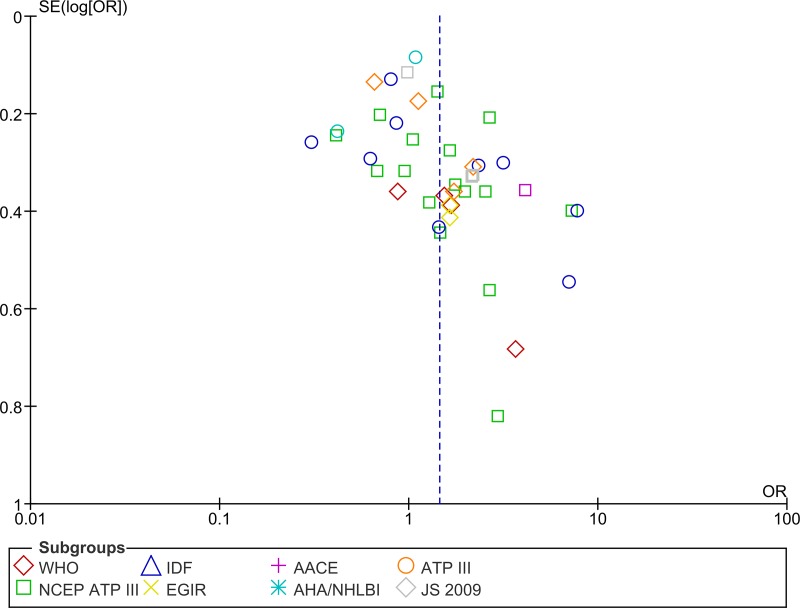
Funnel plot of MetS risk among RA Patients by definition/criteria.

In order to assess publication bias in relation to the OR for MetS and RA, funnel plots and Begg's correlation were used. These found no evidence of any publication bias (Fig 7).

### Meta-regression

To assess the sources of heterogeneity, four variables were included in a univariable meta-regression. Our results indicated that the study date (P = 0.60) and country (P = 0.38) were not responsible for the heterogeneity in the ORs for MetS in RA patients, compared to healthy controls, but mean age (P = 0.03) and diagnostic criteria (P = 0.04) could be considered sources of heterogeneity. Hence, subgroup analysis was undertaken based upon the diagnostic criteria.

## Discussion

The present study found a MetS prevalence of 30.65% among RA patients, but this rate ranged from 14.32% to 37.83%, depending upon the MetS definition used. The relatively high degree of variability in MetS prevalence, according to the MetS definition used, is clearly a substantial issue that permeates the literature on this topic. For example, research in Asia has reported the prevalence of MetS to be 45.2% among RA patients using the NCEP-ATP III criteria [21] and 19.6% when using the WHO definition[22]. In Europe the prevalence rates reported, according to criteria used were: AHA (27.4%), IDF (35.2%), IDF-AHA (37.2%) and NCEP-ATP III (23.0%)[23]. Furthermore, based on the NCE-P-ATP III criteria, Oliveira et al. found that the prevalence of MetS among RA patients in South American was 51.4%, but using the IDF criteria this proportion was 53.4% [24]. Much larger differences have been reported in research from the UK, with MetS prevalence ranging from 8.2% to 42.6% [25], depending upon the definition used. Moreover, in a cross-sectional study which used three definitions (NCEP-ATP III, IDF and AACE) the prevalence of MetS in RA patients varied from 24.6 to 30.7% [26]. Finally, the results of a case- control study in 2013 showed that the frequency of MetS in RA patients and the control group were 30% versus 39% (respectively) when using the ATP III definition and 35% versus 40% (respectively) when using the IDF [27] definition.

Therefore, it appears that some of the variation in the prevalence reported are to do with i) a lack of definition clarity, with many different criteria in the existing definitions, ii) different and multiple phenotypes included in each definition of MetS, and iii) the lack of consistency in the number of components required by each definition.

However, prevalence rates also vary widely even when comparing studies that have used the same criteria. For example, using the NCE/ATP definition, Dessein et al. reported a MetS prevalence of 19% among 74 RA patients [28], while a separate study using the same definition reported a prevalence rate of 42% in those with long standing RA and 30% in those recently diagnosed with RA[29]. Further, in a study of 107 female RA patients a MetS prevalence of 18.7% [30] was reported, but using the same definition Crowson et al. reported the prevalence to be 33%[31]. Therefore, it is likely that other factors related to the characteristics of the study population, such as: genetic, ethnic, cultural, demographic, socioeconomic and clinical factors, also affect the prevalence. Thus, studies conducted using different populations are critical in order to identify other factors related to MetS.

In this study the risk of MetS in RA patients was 45% higher than that in the healthy control group (OR = 1.45; 95% CI: 1.20–1.75). The OR found in the present study is considerably higher than that reported in a meta-analysis of 12 studies in 2013, which reported an OR of 1.24 (95% CI, 1.03–1.50) [11]. Furthermore, Karvounaris et al. found prevalence of MetS to be similar in RA patients (44%) to their control population (41%), but they also found a relationship between disease activity and the presence of MetS [32]. It is also worth mentioning that several studies have not reported any association between RA and MetS [33, 34].

When we assessed the individual components of MetS (FBS, HDL, BP, Triglyceride, WC), a high WC had the highest prevalence, while the lowest prevalence was high FBS. These findings are consistent with a cross-sectional study by Zafar et al., which found that high FBS (21.9%) was the least prevalent component, while a high WC (46.1%) was the most prevalent component[35]. Furthermore, a study of 200 rheumatoid arthritis outpatients reported that the prevalence of a high WC was 74.8% in female patients and 60.4% in male patients, while the prevalence of high FBS were 30.6% and 26.4% in female and male patients, respectively [32]. In another study, blood pressure, hypoglycemia and HDL had prevalence’s of 35.9%, 22.95 and 68.9%, respectively [36]. Therefore, it seems that in most studies a high WC is the most prevalent MetS component and targeting preventative measures at this may considerably reduce the risk of developing MetS.

## Advantages

The present study has a number of advantages over the previous meta-analysis, including: 1) All of the published studies were included in this meta-analysis. 2) The prevalence of metabolic syndrome was investigated in RA patients from across the world. 3) This study reported the prevalence of MetS in RA patients based upon eight separate definitions. 4) This paper included both comparative cross-sectional and cross-sectional studies. 5) The odds ratio for metabolic syndrome was pooled across a large number of studies.

## Limitations

1) Several countries have not assessed the prevalence of MetS in RA patients and therefore data from those countries could not be presented in this study. 2) The crude (unadjusted) odds ratio for MetS in RA patients was reported, as different studies used different set(s) of confounders.

## Conclusion

The prevalence of MetS in RA patients was relatively high, but did not vary significantly by gender. According to the high prevalence of MetS in RA patients and the high risk of it, monitoring and testing for metabolic syndrome in these patients is clearly recommended. As the most important component of metabolic syndrome was found to be a high WC, it is clearly important to pay more attention to patient nutrition and weight loss. Finally, mean age and the diagnostic criteria used to diagnose MetS were identified as sources of heterogeneity in the estimated risk of MetS.

## Supporting information

S1 Appendix (TIFF)

S2 Appendix (TIFF)

S3 Appendix (TIFF)

S4 Appendix (TIFF)

S5 Appendix (TIFF)
