# Supplementary figures and images for: Metabolic syndrome and its components among rheumatoid arthritis patients: A comprehensive updated systematic review and meta-analysis
Source: PLoS One. 2017 Mar 23;12(3):e0170361. doi: 10.1371/journal.pone.0170361 (PMC5363810; doi:10.1371/journal.pone.0170361)

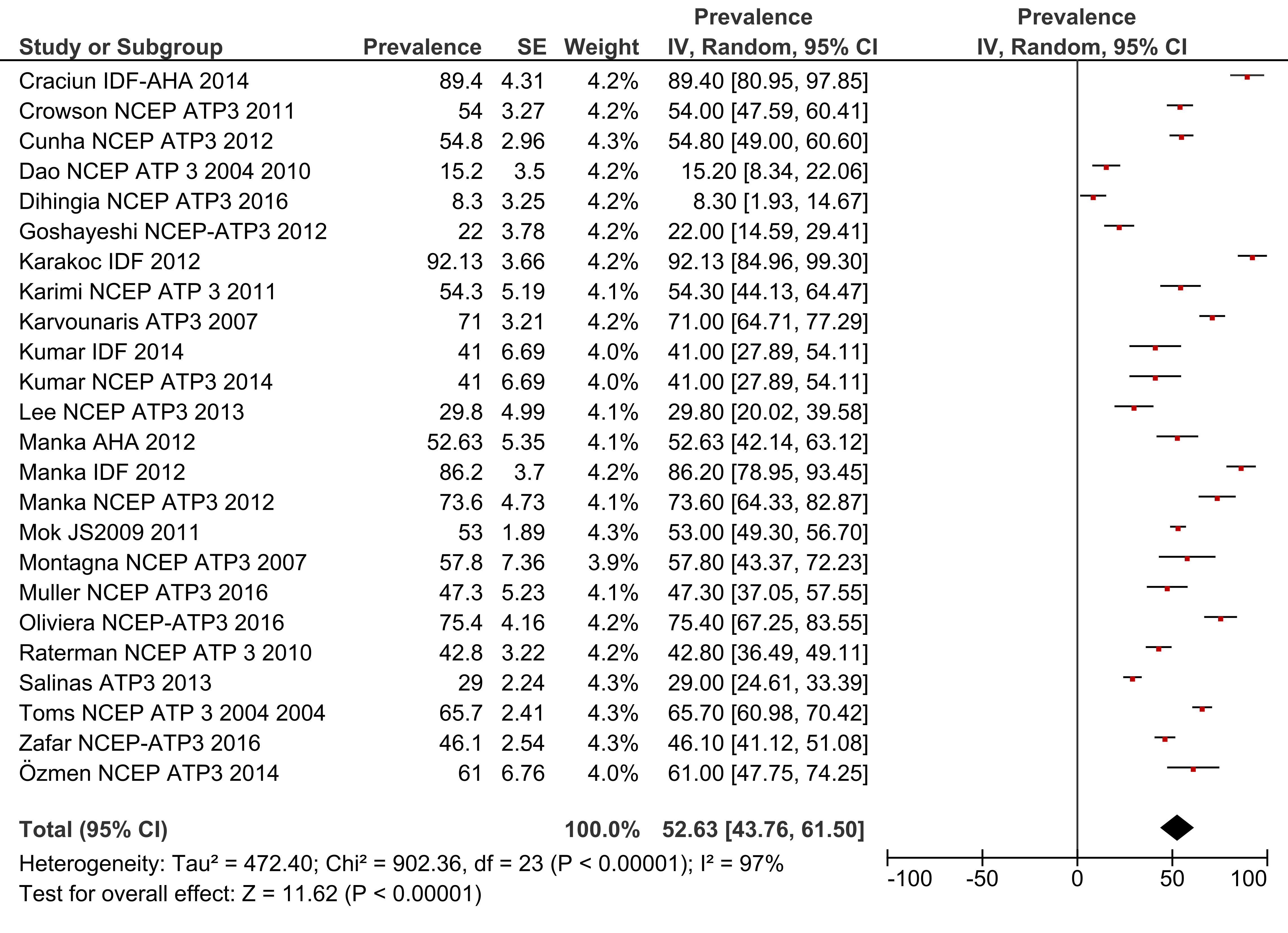

Supplement: S1 Appendix — (TIFF) [file pone.0170361.s001.tiff]

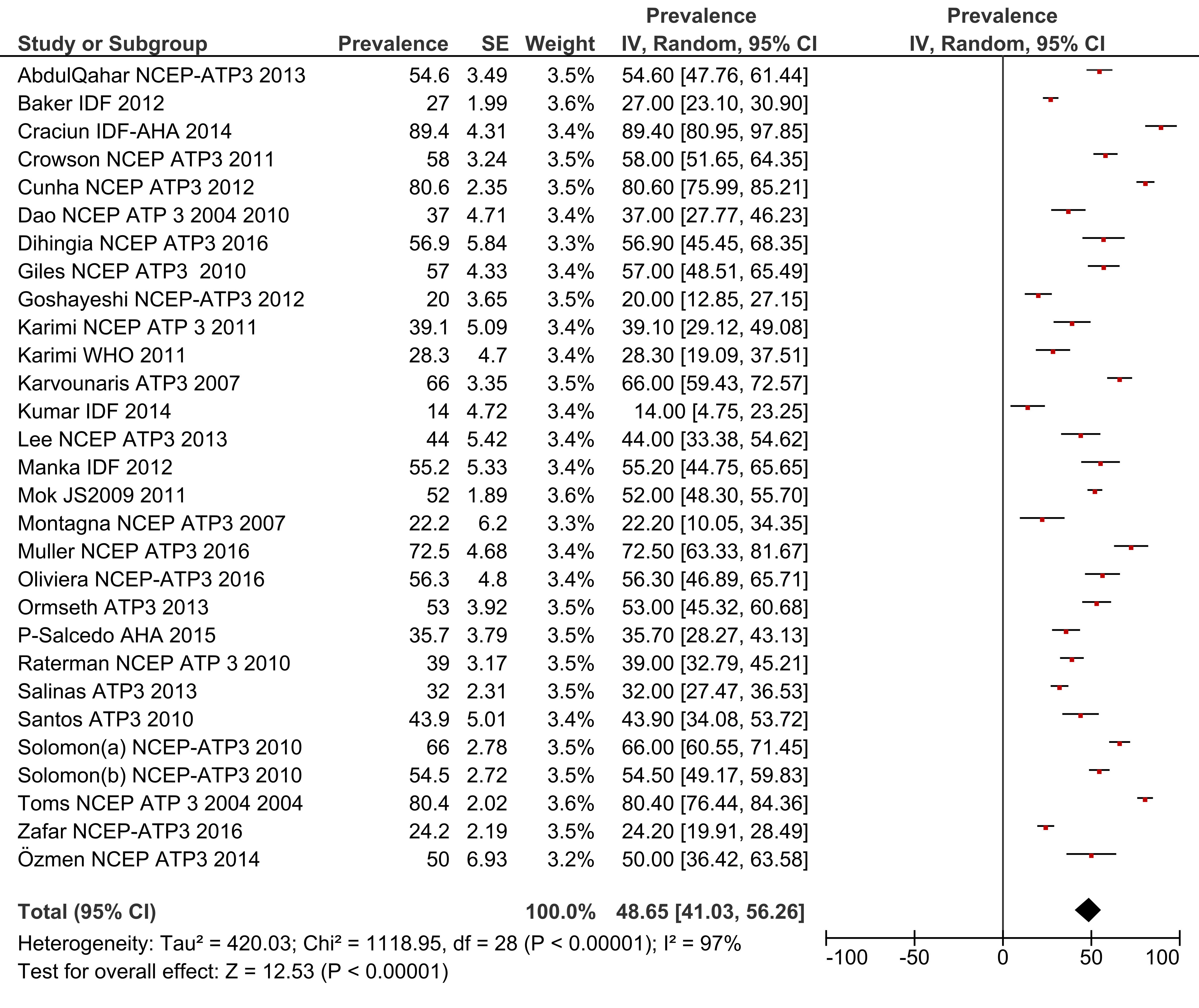

Supplement: S2 Appendix — (TIFF) [file pone.0170361.s002.tiff]

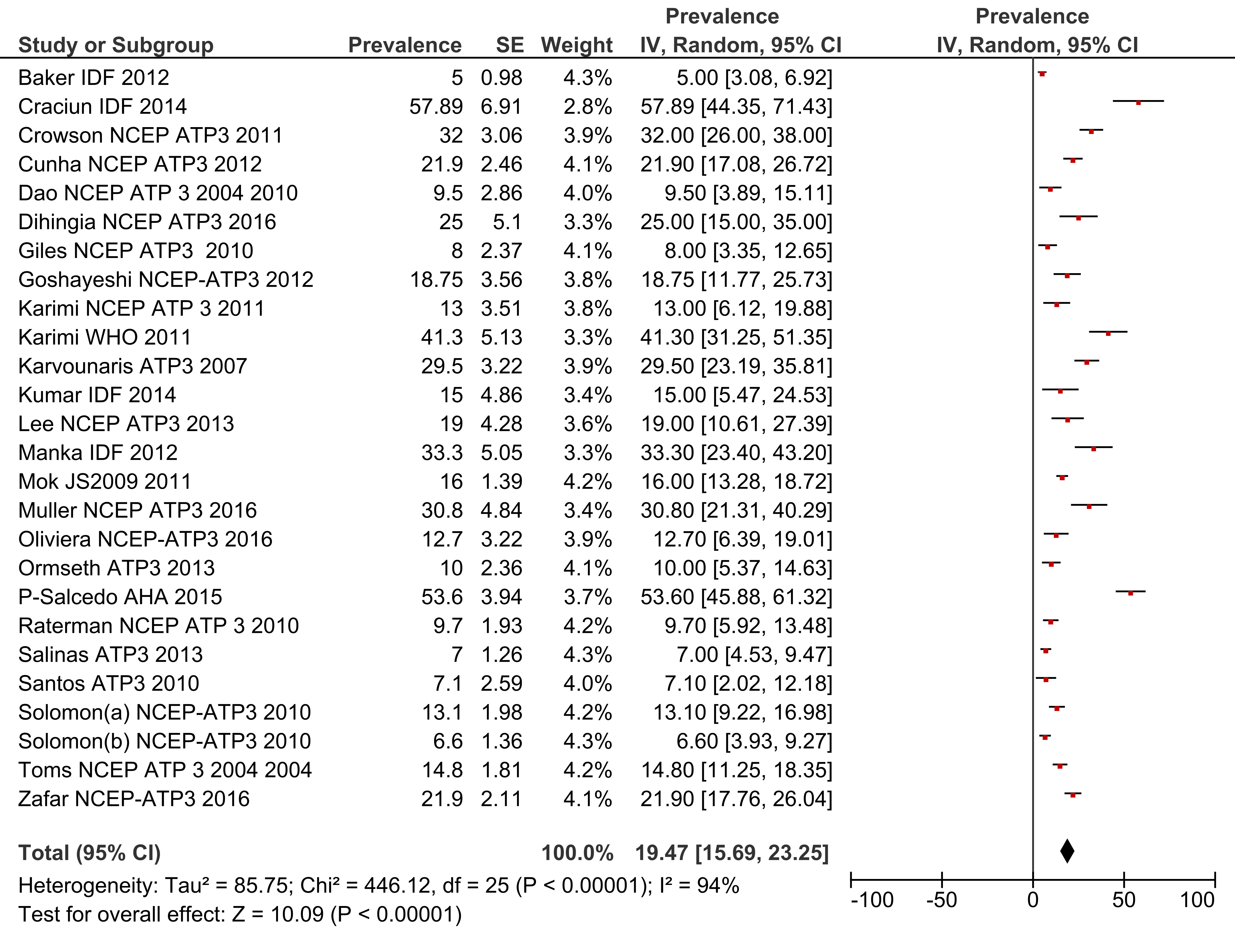

Supplement: S3 Appendix — (TIFF) [file pone.0170361.s003.tiff]

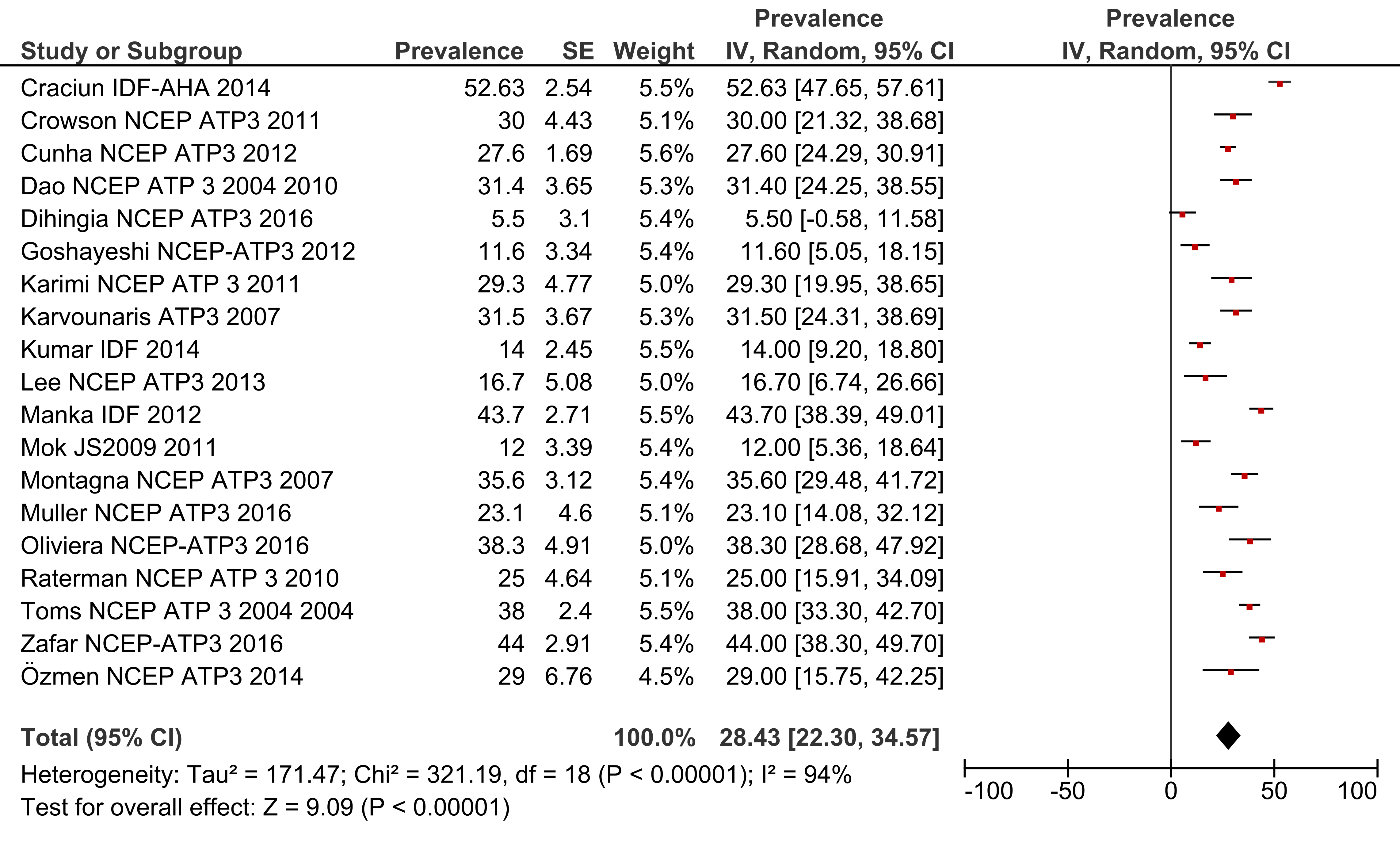

Supplement: S4 Appendix — (TIFF) [file pone.0170361.s004.tiff]

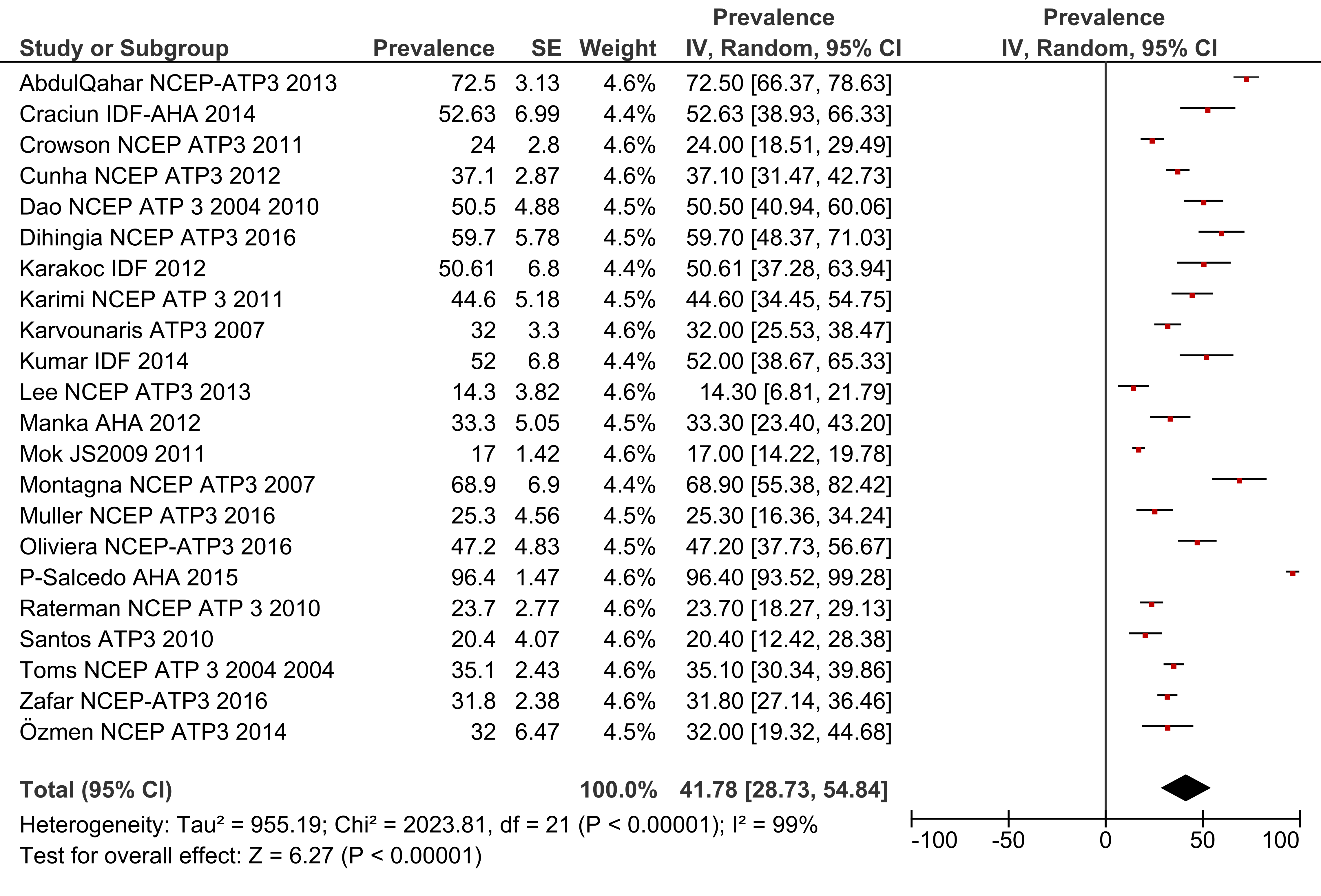

Supplement: S5 Appendix — (TIFF) [file pone.0170361.s005.tiff]
